# First Parent-Child Meetings in International Adoptions: A Qualitative Study

**DOI:** 10.1371/journal.pone.0075300

**Published:** 2013-09-25

**Authors:** Aurélie Harf, Sara Skandrani, Rahmeth Radjack, Jordan Sibeoni, Marie Rose Moro, Anne Revah-Levy

**Affiliations:** 1 Maison de Solenn-Maison des adolescents, Hôpital Cochin, AP-HP, Unité INSERM 669, Université Paris Descartes, Sorbonne Paris Cité, Paris, France; 2 Centre de Recherche Didier Anzieu, Université Paris Ouest, Maison de Solenn-Maison des adolescents, Hôpital Cochin, AP-HP, Paris, France; 3 Centre de Soins Psychothérapeutiques de Transition pour Adolescents, Hôpital d'Argenteuil, Unité INSERM 669, Université Paris Descartes, Sorbonne Paris Cité, Paris, France; The University of New South Wales, Australia

## Abstract

International adoptions involve approximately 30000 children worldwide each year. Nearly all of the adoptive parents travel to the child's country of birth to meet them and bring them home. The objective of this study is to analyze the adoptive parents' account of their first meetings with their child. The study includes 46 parents who adopted one or more children internationally. Each parent participated in a semi-structured interview, focused on these first parent-child meetings. The interviews were analyzed according to a qualitative phenomenological method, *Interpretative Phenomenological Analysis*. The principal themes that emerged from the analysis of the interviews were: the scene when the child is entrusted to the parents, the discovery of the child's body, and the first parent-child interaction. Within these three principal themes, several subthemes dealt with difficult experiences: moments of solitude and anxiety, shocking images of the children's living conditions, lack of preparation and of information about the child, poor health, parental reactions of rejection, worry about the child's body, aggressive reactions by the child, worry about the child's reactions, and contrast with the expected interaction. Thirty-two interviews included at least one of these subthemes. At the structural level of the discourse; the characteristics of 33 interviews are those described in the literature as significantly more frequent in traumatized than in non-traumatized subjects. These results raise questions about the consequences of difficult, possibly traumatic experiences, at the moment of meeting the child, and they underline the need for work on preparation and prevention before the parents leave on their journey.

## Introduction

International adoption refers to the legal adoption of children born in foreign countries. Although we will be discussing specifically such adoptions by French citizens, worldwide international adoption involves more than 30000 children a year, moving between more than 100 countries [1]. Most research in the field of international adoptions has studied the adoptees and the potential impact of their living conditions before adoption on their psychosocial development: they have often endured inadequate prenatal and perinatal medical care, maternal separation, psychological deprivation, inadequate health care, and neglect, abuse, and malnutrition in orphanages or poor families before their adoptive placement [2]. In addition, a part of their lives is missing, and, like all adopted children, they must cope with the fact that they were given up by their biological parents. Two meta-analyses have concluded that international adoptees with extremely adverse preadoption placement history have more behavior problems than international adoptees without such histories and than non-adopted control children [3], [4].

When studies have looked at the adoptive parents, it has been to question them about the child's current problems [5], [6] or to explore the bonds they did or did not decide to maintain with the culture of the child's country of birth [7]. The point of view of adoptive parents has also been explored in studies to examine the question of the child's cultural identity or ways to teach the child strategies to deal with racism and discrimination [8]. Some authors mention the adoptive parents' long and difficult route to adoption: most adoptive parents underwent stressful medical treatments for infertility and then waited several years before having the opportunity to meet a child they could adopt [9].

On the other hand, to the best of our knowledge, no study has explored the adoptive parents' feelings and experiences at their first meetings with their child, during their trip to the child's country of birth.

The purpose of the present study is to explore, from the parents' point of view, the meeting between the adopted child and his or her parents in an international adoption. Our objective was to provide guidance for counselors working with adoptive families.

Because little is known about the parents' experience on their first meetings with the child, we approached this research question qualitatively. Qualitative study is recommended when the goal is to uncover the common and unique experiences of individuals who have firsthand knowledge of the phenomenon of interest [10], [11].

## Methods

### Participants

Eligible participants adopted at least one child from a country other than France. The sample comprised 46 adoptive parents who volunteered to participate in the study, 12 fathers and 34 mothers. Eleven parents had two internationally adopted children and were interviewed twice (one interview for each child). We thus had a total of 57 interviews. Data saturation determined the sample size.

Overall, 32 parents were married and 14 had adopted as single parents. In 11 families both parents participated. Parents' ages at the time of their children's adoptions ranged from 28 to 49 years and at the time of the interview from 31 to 60 years. Parents lived in urban areas of France. Most (86%) were college-educated professionals.

Of the 42 internationally adopted children, 23 were girls and 19 boys. At the time of their adoption, their ages ranged from 2 weeks to 7 years. Fifteen children were younger than 1 year at the moment of their adoption, 11 were between 1 and 2 years old at adoption and 16 were older than 2 years. One child was adopted in Algeria, 1 in Armenia, 2 in Bulgaria, 1 in Brazil, 2 in Cambodia, 1 in the Central African Republic, 3 in China, 1 in Colombia, 1 in Ethiopia, 1 in Guatemala, 7 in Haiti, 1 in India, 3 in Lithuania, 2 in Madagascar, 1 in Mali, 2 in Poland, 2 in Romania, 1 in Russia, 2 in Thailand, and 7 in Vietnam.

At the time of the interview, the children's ages ranged from 15 months to 17 years. The time between the adoption and the interview ranged from 1 year to 16 years: of the 57 interviews, 14 concerned children adopted within two years of the interview, 11, 2 to 5 years before the interview, and 32, children adopted more than 5 years earlier. Nine families had both adopted and biological children. The number of children per family ranged from 1 to 4, and the number of adopted children from 1 to 2.

An effort was made to vary the sample in terms of age, life stage (i.e., families with young children as well as those with adolescents), and family structure (single parents and married couples). Sampling in qualitative research involves purposive sampling of individuals liable to provide the most informative description of the phenomenon under study [12]. Our sampling technique was indeed purposive, because we selected subjects who were typical of the population of interest [13].

The participants were recruited in the general population through adoption associations and connections between adoptive parents.

We note that there were several parent-child encounters before the adoption, during different trips, for 5 children, from Russia, Bulgaria, and Romania, because of legal requirements in those countries. For the others, the process was completed in a single journey: either parents left with the child at the end of the first meeting (29 children), or there was one meeting and then the parents returned the next day or the day after to pick up the child (8 children).

### Data Collection Procedure

Data were collected via semi-structured interviews. The authors reviewed the international adoption literature to develop a guide for these parental interviews. The broad topics covered included the choice of country, the trip to the child's native country, and the first interactions with the child. The interview protocol that guided this research is included in the appendix (Appendix S1).

Questions were designed to obtain specific information while remaining flexible so that the interviewees could tell their stories. Open-ended questions allowed participants to interpret the meaning of the question and respond according to their personal feelings. Interviewers used prompts and probes as needed to enrich the discussion. We chose to collect data through semi-structured interviews because this method combines an approximate standardization of questions with the opportunity for subjects and interviewers to expand their answers when appropriate. The interviewing process produced deep and broad data that focused on the research question: how subjects described their experience of their first meetings with the child they were adopting.

Parents chose the interview site: the researcher's office or their own home. The length of the interviews was determined by the participants and what they had to say; they averaged one hour. Every interview was audio-taped for later transcription, with the participants' permission, and transcribed verbatim in French. Two different researchers conducted these interviews, separately. Each had training in the fields of adoption and qualitative research methods (AH, SS).

### Data analysis

A phenomenological research design was employed to understand the lived experience of parents who went to their child's country of birth. Phenomenology is a nonprescriptive approach to research that allows the essence of experience to emerge, yet anchors data analysis in the participants' unique representations [13]. The aim is to explore personal experience, and the subjective perception of an object or event. Our research approach is phenomenological in that it involves detailed examination of the participants' personal perceptions and lived experiences. To analyze our interview data, we used the *Interpretative Phenomenological Analysis (IPA)* method [14], [15], an established qualitative methodology used to explore in depth how individuals perceive particular situations they are facing and how they are making sense of their personal and social world [16]. Following the *IPA*, we conducted an in-depth qualitative analysis. It began with a detailed case-by-case study of each interview transcript, according to an iterative inductive process. We began with several close detailed readings of each interview to provide a holistic perspective, noting points of interest and significance. Through a step-by-step analysis, we proceeded to the description of analytic themes and their interconnections, while taking care to preserve a link back to the original account. Doing *IPA* thus involves navigating between different levels of interpretation [14]. The last stage involves the production of a coherent ordered table of the themes [17]. The procedure for data analysis was inductive as the analysis of data from the literature was performed secondarily. The size of the sample was determined by data saturation, that is, the point at which in-depth analysis of the interviews no longer results in the emergence of new themes. We had no preconceived ideas of the number of patients to include, but the analyses enabled us to confirm that we had reached data saturation.

Throughout, we employed computer software to assist our analysis: QSR NVivo for data management, topic extraction from and thematic recodes of individual interview transcripts.

### Validity

To insure the validity of our qualitative research, we compared the researchers' coding. Two trained researchers (AH and SS) independently coded and interpreted all of the parent interview data. The two coders discussed the emerging codes in repeated meetings with other members of the research team (ARL and MRM) who had read the transcripts. These discussions helped to identify potential themes in the data that might not yet have been captured by the codes, and enabled us to clarify or modify the coding to increase the consistency and coherence of the analysis by ensuring that the themes identified accurately reflected the data and that the analysis was not confined to one perspective. Multiple discussions allowed us to eliminate systematic differences due to variations in interpretation. Validity was also enhanced by the care we took to distinguish clearly between what respondents said and how we interpreted it or took account of it [17].

Member-checking (also known as informant feedback or respondent validation) was practiced, for it is a vital way for interpretive researchers to verify the trustworthiness of their research [18], [19]. When the qualitative analysis of the parents' data was completed, a summary of the thematic results was mailed to the parents, who were asked to provide feedback, reactions, and comments. Participants were asked to share these preliminary results with spouses who had been unable to take part in the interview. Ten of the 46 parents provided written or verbal feedback, which was incorporated in the final results. This methodological aspect of the study provided a source of testimonial validity [20] for the qualitative results and enabled us to take participant feedback into account in their interpretation [21] and to assess the degree to which the themes resonated with the parents' experience.

### Ethics statement

Parents were fully informed of the voluntary nature and the goals of the study. Written informed consent was obtained from all parents included in the study before the interview. Participants were informed that all responses would be confidential, that the transcripts would have no identifying information, and that they would be free to withdraw at any time. All identifying information was removed from the transcripts, and participant anonymity further ensured by disguising or withholding of descriptive data. The Ethical Review Committee (Institutional Review Board of Paris North Hospitals, Paris 7 University, AP-HP, N° IRB00006477) approved this research protocol.

## Results

Phenomenological analyses of the interviews yielded three salient aspects of the parents' experiences at these first meetings: the scene when the child is officially entrusted to the parents and leaves with them, the discovery of the child's body, and the first parent-child interaction. We also present the results of the structural analysis of the interviews.

The results below include excerpts of respondents' verbatim accounts, chosen to exemplify the recurrent underlying themes. To protect confidentiality, identifying information has been removed from the quotations presented. The verbatim account has been freely translated into English for the sole purpose of this article; the main objective of the translation is to preserve the essential meaning, content, and insofar as possible general tone. Ellipses within brackets indicate the deletion of a part of the sentence. For greater clarity and to preserve the parents' anonymity, parents are designated as Mothers 1 to 34 and Fathers 1 to 12. The child's age at the time of the interview has been added to the end of each quotation, following the parent's number (e.g., Mother 5, of a 6-year-old).

Table 1 details the number of subjects concerned for each of the subthemes. The themes we identified were replicated across families, despite differences in the children's ages at adoption.

**Table 1:** Themes and subthemes extracted from the analysis of the interviews.

			Number of fathers	Number of mothers	Number of parents
**Scene in which the child is officially entrusted to the parents**	Emotional coloring	Magic moment	1	9	10
		Moment of solitude and anxiety	3	15	18
	Images of the orphanage	Shocking images of the children's living conditions	6	17	23
	Adults present	Lack of preparation and of information about the child	2	12	14
**Discovery of the child's body**	Description of the child's physical condition	Poor health status	5	17	22
	Parents' emotions aroused by the child's body	Parental reaction of rejection	2	7	9
		Worry about the child's body	3	12	15
**First interaction between the parents and child**	The child's reactions	Accept contact	4	6	10
		Aggressive reaction by the child	6	8	14
	Parental emotions in response to the child's reactions	Happiness	2	7	9
		Worry about the child's reactions	4	6	10
		Contrast with the expected interaction	0	8	8
	Parents' explanations for the children's reactions		1	8	9
**Structure of the discourse**	Incomplete sentences		6	6	12
	Passage from one idea to another without any logical association		6	10	16
	Absorption in a scene from the past with passage from the past to the present		4	14	18
	Reduction in the vocabulary attached to emotions or feeling		7	15	22
	≥1 of the four characteristics described above		8	25	33

### Being entrusted with the child

The first theme concerned the handover of the child to the parents, that is, the final leave-taking of the orphanage. Three subthemes emerge: the emotional coloring of the scene, the images of the orphanage, and the lack of preparation and information of the adults present.

#### Emotional coloring: a magic moment or a moment of solitude and anxiety

In 10 interviews, this scene of the meeting was mentioned as a magic moment, intended by fate, bonding at first sight, mutual recognition. The strength of the parent's emotion was stressed.

*“The most beautiful memory is when I went into the orphanage, when i saw the director on the right, and then, in the back, I saw a volunteer with, with my baby, ah, and then, oh that was it! It was like a punch in the heart. I have never experienced that in my life, like, that was a moment. Here, I had tears in my eyes because it was something, uh, something marvelous, really, marvelous”* (Mother 4, of a 7-year-old).

*“I saw him in front of me. I don't know how to express it, it seemed to me outside of time, a moment that was really separate, extraordinary, when we met”* (Mother 23, of a 4-year-old).

On the other hand, in 18 interviews the feeling of solitude was highlighted by the parents, a feeling of uneasiness and anxiety.

*“It was a little strange (…) the director of the orphanage was watching me, I had the feeling of doing wrong, uh, of not knowing what to do (…) the memory is very uh… I was very ill at ease in fact during this meeting”* (Mother 2, of a 17-year-old).

*“I was really anxious, I would say”* (Mother 10, of a 2-year-old).

*“You take your bag and you leave (…) You go back to the hotel, and you find yourself alone”* (Mother 17, of a 3-year-old).

Of these 18 parents, 6 reported that the birth mother was present at this meeting. Four of these 6 mothers described a great sense of unease during the meeting with the birth mother. There were no interpreters, and the conversations between the two mothers were very limited. The fifth adoptive mother, on the other hand, was reassured to see that the birth mother was pretty and "normal". Finally, the sixth adoptive mother to meet the birth mother described a very negative experience of the handover of the child in the birth mother's presence.

*“It's a violent way to receive a child (…) it was violent this way of seeing the biological mother. I had the impression that she had put him in my arms, but that she might take him back (…). I felt awful, I had the impression that I'd stolen him, and worse, that they might take him back from me”* (Mother 18, of a 16-year-old).

Nonetheless, no emotion was expressed in more than half the interviews (n = 29): the parents said nothing about their emotional experience of this very special moment when they were entrusted with their child. In those cases, the parents' accounts contained only a very detailed description of the context and the place where the scene took place.

#### Images of the orphanage

In 23 interviews, the description of the meeting place and of the children present in the orphanage occupied most of the parents' accounts. The images were shocking to the parents. The first meeting with the child was associated with an intense confrontation with extreme poverty. *“It was awful. All those children who clung to us, who hung on to us (…) an awful thing”* (Mother 8, of a 6-year-old).

*“They were lying in pee and poop all the time; that's why they didn't want us to leave a stuffed animal, because of the hygienic conditions (…) They ate alone, they were given a bottle and then as soon as they could, a bowl with gruel but the bigger children took it away from them, so they didn't eat much”* (Mother 14, of a 7-year-old).

*“There was a very large park, very nicely equipped, with pretty trees and all sorts of children: children left there because without they had no arms, no legs, they moved by swimming on the ground, horrible, then practically in the same place, because they were wandering between the beds in the hallways, little beds, and in each bed two babies. I still can see her there, sad, attached to her thing, wanting to grab me. (…) When you arrive in this universe, you're not used to it, especially the deformed children who are close by, who also wanted something”* (Father 3, of a 16-year-old).

Four parents adopted children right after the January 2010 earthquake in Haiti. These parents went to the orphanage directly from the airport, and the moment that the child was entrusted to them remains associated with very powerful images – of a country that had just undergone an earthquake, of orphanages partially destroyed.

#### The adults present: absence of preparation and information about the child

One particular point emerged from the analysis of 14 interviews: the orphanage staff present did not appear to have prepared for the meeting or to have information about the child to provide to the parents. Although the director or caregivers or both were there, the parents said that they had not talked – or very little – about their child, his or her history, temperament, or habits. The parents thus described a child handed to them abruptly and about whom they knew nothing. The parents emphasized the contrast between the length of the wait to be allotted a child and the speed with which the child was turned over to them.

*“Bang, the baby is in our arms; he was ours right away, from one moment to the next.”* (…) *“The procedure went so fast, we were completely unprepared (…) They said to us: come tomorrow with clothes, you'll leave with the baby”* (Mother 8, of a 6-year-old).

*“No time to ask much at all”* (Mother 20, of a 3-year-old).

*“They showed us the child and they gave him to us naked, they didn't even give him the towel he was wrapped in. There was no approach to the child”* (Mother 27, of a 2-year-old).

### Discovering the child's body

The second theme concerned the discovery of the child's body. That is, even when parents received photographs of the child before the meeting, it was only during the first meeting that they discovered what their child looked like. Two subthemes emerged: the description of the child's physical condition, and the emotions felt by the parents seeing the child's body.

#### Description of the child's physical condition

Parents in 22 interviews described in detail their children's health problems at the time they met. Mentioned most often were the consequences of nutritional deficiencies, as well as delays in both growth (weight and height) and psychomotor development. The child's physical appearance could also be shocking because of, for example, skin diseases or purulent otitis. Parents sometimes feared that the child's life might be in danger.

*“She began then, finally, to have a distended belly, uh, flabby, discolored hair”* (Mother 9, of a 4-year-old).

*“Timothy had a major ear injection, with an ear that was running enormously, (…) and then he had an eye, uh, that couldn't see at all”* (Father 6, of a 9-year-old).

*“His back was all black, but really, the whole back as if it were bruised, all black, and the knees all black”* (Mother 22, of a 15-year-old).

*“He just barely sat up, he was a little weak”* (Mother 1, of a 5-year-old).

*“She was tiny, preterm, not quite 4 pounds, there wasn't much hope for her, (…) Her temperature was low, she hardly ate, her temperature was falling”* (Father 2, of a 2-year-old).

#### Parents' emotions aroused by the child's body

In the interviews, some parents described their feelings about the child's poor health at the moment of the first meeting. Nine mentioned, with a great deal of guilt, their reaction of shock, withdrawal, or rejection of the child at that moment, related to his or her physical condition.

*“I stepped back, withdrew, as soon as I had the child, I withdrew, uh. It was a tiny baby, puny, sickly, not in very good shape”* (Mother 19, of a 2-year-old).

*“It was horrible and I didn't see myself with this sick child”* (Father 6, of a 9-year-old).

The child's poor health also led to parental worries about the consequences of the living conditions before the adoption, in terms of the child's subsequent physical and psychological development. This poor health thus testified to the inadequate care the child had received before the adoption. Fifteen parents reported that this worry was the dominant emotion at the first meeting.

*“He arrived at the orphanage; he was not very well fed or well cared for… I understood that the mother had not been there for several days. The police brought them in as an emergency, alcoholism, maybe drugs”* (Father 5, of a 7-year-old).

*“It was a child that was put there, just like that, that we were taking, who hadn't been cared for, and I think that's what's made him what he is now”* (Mother 10, of a 2-year-old).

### Description of the first parent-child interaction

The third theme is the description of the first parent-child interaction. We distinguished three subthemes: the child's reactions, the parent's emotions in response to the child's reactions, and the meaning that the parents attributed to the child's reactions.

#### The child's reactions

In 10 interviews parents described a child who immediately moved toward them and accepted contact.

*“And then I took her in my arms and then, uh, she trembled and then, I don't know. Uh, ten minutes after, she was sleeping on my lap, and it's the most beautiful memory that I have, because, in fact, she adopted me in those ten minutes. I think it was the most beautiful day of my life”* (Mother 4, of a 7-year-old).

*“He gave me his hand and looked at me”* (Father 3, of a 16-year-old).

On the other hand, parents in 14 interviews described fierce reactions of rejection by the child at the first meeting. The children could be agitated, uncontrollably angry, aggressive toward themselves or to others. The 14 interviews in which parents described an aggressive reaction by the child all involved children adopted after their first birthday.

*“She expressed herself violently enough (…) She decided not to get out of the car. She wanted to stay, so, she started to cry, to scratch my husband who was trying to get out of the car (…) It was the evening, we finally persuaded her to get out of the car, that took a long time, and then she had another fit in front of the building where we had the apartment and then she began to scream, but for …I don't know.…a long time, shrill screams”* (Mother 25, of a 11-year-old).

*“The people at the orphanage had always said she was a little girl who was very sweet, uh, but really sweet and gentle, and then all of a sudden I find myself in front of a little girl who's hitting me, spitting at me, scratching me”* (Mother 7, of a 8-year-old).

*“He cried, yelled, struggled”* (Mother 3, of a 6-year-old).

#### Parental emotions in response to the child's reactions

When the child accepted the bond with the parents from the beginning, the interviews stressed intensity of the parent's happiness. This was the case for 9 interviews.

*“Magic”* (Mother 24, of a 4-year-old).

*“Marvelous”* (Mother 4, of a 7-year-old).

On the other hand when parents described reactions of rejection or violence by the child at the first meeting, they stressed that they were not expecting such reactions. In 10 interviews, the first moments spent together were a worrisome experience.

*“She yelled so loud that after her eyelids were all red, and then, I was very scared”* (Mother 25, of an 11-year-old).

*“It was hard, I didn't know what to do, she didn't want me, and it didn't stop”* (Father 4, of a 6-year-old).

*“I remember that I was very worried because he was doing a thing like that, he had [a small car with] wheels and he turned them and he was in this world of wheels that were turning and I wasn't in that world”* (Mother 6, of an 11-year-old).

Eight mothers explained that what they found difficult in this first interaction with their child was that it wasn't the way they'd imagined it.

*“It was really very hard because she did not resemble the baby that I had idealized”* (Mother 19, of a 2-year-old).

*“The beginning was hard because I think you completely idealize a child and then you have him in your arms all of a sudden and, well, it's not as obvious as all that”* (Mother 27, of a 2-year-old).

#### Parents' explanations for the children's reactions

Among the parents describing reactions of rejection by their child at the first meeting, 9 tried to find explanations for the child's reactions.

*“Clémentine was afraid; she didn't know what was happening to her. We were each feeling equally disoriented by the other”* (Mother 16, of an 8-year-old).

*“I think a lot must have gone on in her head…changing countries, changing friends, I think they were some real dislocations in her life”* (Father 4, of a 6-year-old).

### Structure of the discourse

The analysis of the interviews showed 4 characteristics specific to the narrative structure:

• In 12 interviews, there were numerous unfinished sentences.
• In 16 interviews, the story passed from one idea to another without any comprehensible logical association.
• In 18 interviews, there were long descriptions, with excessive attention to details, which gave the impression that the subject was absorbed in scenes of the past. The register of the vocabulary was essentially sensory. The parents thus used the present tense while they were recounting the meeting, while the rest of the interview was in the past tense.
• In 22 interviews, there was a reduction of references to inner experience, and the expression of feelings was very limited. The meeting scene was described as if viewed from outside, without words indicative of emotions or feelings.

In conclusion, in terms of discourse structure, 33 interviews had at least one of the following four characteristics: incomplete sentences, passage from one idea to another without any logical association, absorption in a scene from the past with passage from the past to the present, and finally a reduction in the vocabulary attached to emotions or feeling.

### Respondent validation

These results were sent by mail to 16 parents, who had agreed in advance to review the results. Ten of them responded. The themes uncovered by the qualitative analysis matched the ways the parents would have described the first parent-child meetings. They emphasize the worry that occurs when the child's reaction is one of rejection, the fact that the staff at the orphanage told them little about the child at the time of the meeting, and the very trying journey they had to go pick up the child. All the parents who responded to this feedback found that these themes were applicable to adoptive parents in general, and they all had other examples of adoptive parents to cite. Interestingly, they did not want to mention what made their own experience difficult; on the contrary, they insisted that they themselves were lucky, in comparison with other parents.

### Synthesis of results

The phenomenological analysis of the parent interviews, which focused on the first meetings with the child during the trip to the child's country of birth, underlined three principal themes: the scene in which the child was officially entrusted to the parents, the discovery of the child's body, and finally the first interaction between the parents and child.

In these three themes, several subthemes called attention to the brutal and difficult character of that first meeting for the parents.

Subthemes including brutal and difficult experiences:

• moment of solitude and anxiety
• shocking images of the children's living conditions
• lack of preparation and of information about the child
• poor health status
• parental reaction of rejection
• worry about the child's body
• aggressive reaction by the child
• worry about the child's reactions
• contrast with the expected interaction.

The analysis showed that 32 of 57 interviews described brutal and difficult experiences for the parents at the moment of the parent-child meeting.

Among the 32 interviews describing such oppressive experiences, 13 involved children less than a year old at adoption, 12 aged from 1 to 2 years, and 7 adopted at the age of 2 years or more. In addition, of the 32 interviews describing oppressive experiences, 8 concerned children adopted less than 2 years before the interview. In 10 interviews, the time between adoption and interview ranged from 2 to 5 years, while 14 concerned children adopted more than 5 years earlier.

Eleven of the parents we interviewed had adopted 2 children. For 4 parents, these were simultaneous adoptions of siblings. The other 7 parents who adopted 2 children did so in 2 separate adoptions, in the same country for 4, and in different countries for 3. When both adoptions took place in the same country, knowledge of the country of birth made the experience of the second adoption less difficult. But when the country was not the same for the two adoptions, difficult experiences could occur during the second as well as the first adoption. The first adoption did not prepare them for the second when it was in a different country.

## Discussion

This study illuminates and calls to the attention of adoption professionals the frequency of grim and difficult experiences for adoptive parents at their first meetings with their child. These results raise the question of potentially traumatic experiences for the parents at these meetings. Post-traumatic stress disorder (PTSD) after childbirth has been explored [22], [23], [24]. But to the best of our knowledge, there is no literature about the potentially traumatic experiences of adoptive parents at the time they meet their child. This hypothesis must be explored when working with adoptive families because the first interactions with the child have major repercussions on the subsequent construction of the parent–child bond [25]. We know that adoptive children arrive with their own traumas, related to their difficult living conditions before adoption [26]. But it is also essential to look for traumatic experiences among the parents. It is a challenge to take in a child who is no longer a baby, who has experienced hostile or difficult living conditions before the adoption. Helping parents to cope with this challenge requires exploring how they experienced their first meeting with the child [27].

The responses by parents who agreed to comment on our results are interesting. We can summarize them accordingly: “I completely agree with the idea that adoptive parents can undergo difficult experiences. I can give examples. On the other hand, I've been very lucky and did not have to face violent experiences.” However, the experiences described by these very parents completely contradict the affirmation that they were not exposed. It is as if these experiences were still too painful for them to be able to recognize them [28].

Finally the results about the structure of the interviews support the hypothesis that for some parents their first meetings with the child were a traumatic experience. That is, we find in 33 interviews at least one of the following structural elements in the parents' discourse, when they talk about the meeting: unfinished utterances, abrupt progression from one idea to another without logical links, very long and detailed descriptions in which the sensory register predominates, giving the impression that the parent is again immerged in the scene from the past, and a reduction in the vocabulary attached to emotions and feelings. These characteristics are found in subjects with PTSD significantly more often than in subjects without PTSD. A 2006 meta-analysis on the topic of trauma narratives in PTSD showed that the narrative of the trauma is structurally dominated by sensory impressions and perceptual characteristics (sensory impressions described as if they were a current experience) [29]. It showed narrative disorganization (confusion, disjointedness) or fragmentation (repetitions, unfinished utterances, and speech fillers) [29]. Several studies have found that narratives of trauma in PTSD often involve spontaneous shifts from past to present tense verbs [30], [31]. There is also a decrease in references to thoughts and feelings, that is, in utterances reflecting internal experiences [32], [33]. Hesse et al showed that the discourse of traumatized subjects is characterized by sudden changes in the register of the discourse, by silences in the middle of a sentence, followed by the continuation of an irrelevant subject, and excessive attention to details [34].

It is therefore relevant to note that in 33 interviews some characteristics of the structure of the discourses of the parents included in this study were similar to the characteristics found in the discourse of subjects with PTSD. This structural finding points in the same direction as the interpretative result, that is, the presence, for more than half of adoptive parents, of difficult or harsh experiences, possibly traumatic, at their first meeting with their child.

We note that difficult experiences are recounted at equal rates in the interviews of parents who adopted recently, those who adopted from 2 to 5 years earlier, and those with adoptions more than 5 years earlier. Adoptive parents describe difficult experiences, even when they took place several years ago. Moreover, these are described with difficulty by the parents, as if they had just happened. Thus, what is striking in this study is the lack of difference between interviews of parents with recent adoptions and those who adopted several years earlier. This result points to parents' possibly traumatic experience of the moment they first met their child. One of the characteristics of the narratives of traumatized subjects is their use of the present tense to recount traumatic events of the past, as if the events were still happening or had only just ended [29].

A parallel can be drawn with the parents of very preterm babies. One study has showed that the narrative and flow of thought of parents recounting the birth of their preterm child is disorganized. The retrieval of the memory of the events surrounding the birth and the first interactions with the child is marked by intrusive memories, by avoidance or by emotional hypervigilance, all specific to a state of stress or trauma [35]. Parents can also undergo difficult experiences at their first encounters with their child in situations other than adoption or preterm birth. Studies have shown the possibility of difficult experiences, including the risk of post-traumatic stress disorder, following neonatal complications [23], [36], or even in the absence of obstetric complications, in association with the parent's subjective experience [37].

Prevention work therefore appears necessary to prepare parents before they leave to find and pick up their child in his or her country of birth. They must be prepared for the experiences they may face, including problems with the child's health or negative reactions or a negative context surrounding the first meeting. It is important to warn parents about the risk of discrepancies, sometimes large, between the child they have imagined and the child they will meet. Several parents underlined their lack of preparation for this meeting. In France, parents receive little preparation before this first encounter. They can join adoptive parent groups, but there is little if any training organized by adoption agencies in France. Indeed, 40% of the international adoptions in France involve individual initiatives, that is, do not go through adoption agencies. Parents thus travel alone to their child's country of birth, without any specific support. This increases their solitude and its consequence is poor parent preparation before their first meeting with the child. In 53 of 57 interviews, parents reported no preparation about how the meeting would unfold, or who would be present. In a majority of cases, parents report that they did not know if their child had been prepared for the adoption and what he or she might have been told.

The question of the child's age and its consequences must be explained to the parents. The 14 interviews in which parents described a reaction of aggression or rejection by the child at the meeting all involved children past their first birthday. The child's age is thus an important factor to take into account when talking about the child's reactions at this first meeting. Parents must learn about the concepts associated with the theory of attachment, so that they know that reactions of terror or rage are explained by the representations of attachment that the children constructed before their adoption [38], [39]. Children adopted after the age of 9 months have already integrated internal operating models of how the people around them respond to their distress and have adapted to it [40]. Fundamental concepts such as stranger anxiety must also be explained to parents, so that they can understand some of their child's fearful reactions, which are normal but can be very troubling for the parents. To be able to endure some of the child's reactions at the first meeting and respond to them, parents must be able to understand them.

### Limitations

Several limitations must be considered.

One is the question of sample bias, that is, whether and how our respondents differ from non-respondents in ways that limit our analysis.

Another potential limitation is the frequency in our sample of parents who had lived difficult experiences before the adoption. Eight of 46 parents reported potentially traumatic experiences before meeting their child; these included a serious disease that may or may not have been responsible for sterility and the death of a child or of their partner. Eight parents had therefore had other difficult experiences before the adoption, which could have influenced the meeting with the child. In addition 4 mothers had a history of depression, before the adoption.

Finally there are probably differences between what the fathers and the mothers experienced during these first meetings. Studies of early interactions can differentiate the experience of mothers and of fathers after their child's birth [41]. It would have been interesting to see whether we could show such differences in this work on first encounters in adoption. One objective might be to continue this research in differentiating in greater detail between fathers and mothers.

## Conclusions

The increasing number of international adoptions makes it necessary to study the conditions in which they take place and their consequences on the parent-child relationship. The first meetings with the child may be quite different from what parents were expecting in their mental pictures of a successful first meeting after their long struggle for adoption.

The analyses show two principal emotions experienced by parents: feeling frightened and feeling alone. These emotions are strikingly similar to how adopted children live their experiences of fear and abandonment. This study thus shows a symmetry in what parents and child may feel – fear and solitude. It is easier, and more common, to imagine fear in the children than in the parents.

This study, however, is from the parents' perspective and explores their experiences of these first meetings. The study of grim, difficult, possibly traumatic experiences, endured by parents at this meeting should be a priority for professionals working with adoptive families. It is essential to provide support to parents before adoption, to offer them specific help and preparation for what they might face during the journey to the child's country and at their first meeting. The objective of this prevention work is to allow a positive construction of the parent–child bond and to minimize distress and subsequent ruptures, for children and for their parents.

## Supporting Information

Appendix S1 **Interview Protocol.**
(DOC)
