# Supplementary material for: First Parent-Child Meetings in International Adoptions: A Qualitative Study
Source: PLoS One. 2013 Sep 25;8(9):e75300. doi: 10.1371/journal.pone.0075300 (PMC3783391; doi:10.1371/journal.pone.0075300)
Supplement: Appendix S1 — Interview Protocol. (DOC) [file pone.0075300.s001.doc]

**Appendix S1: Interview Protocol**

In what country did you adopt your child?

How old was the child when you adopted him/her?

What memories do you have of the trip?

What do you know about your child's life before the adoption?
